# Shear Wave Imaging of Passive Diastolic Myocardial Stiffness: Stunned Versus Infarcted Myocardium

**DOI:** 10.1016/j.jcmg.2016.01.022

**Published:** 2016-09

**Authors:** Mathieu Pernot, Wei-Ning Lee, Alain Bel, Philippe Mateo, Mathieu Couade, Mickaël Tanter, Bertrand Crozatier, Emmanuel Messas

**Affiliations:** a Institut Langevin, Ecole Supérieure de Physique Chimie Industrielles de Paris, ParisTech, Paris, France; b CNRS, Unité mixte de recherche 7587, Paris, France; c Institut National de la Santé et de la Recherche Médicale, U979, Paris, France; d Université Paris Diderot-Paris7, Paris, France; e Hopital Européen Georges Pompidou, Paris, Unité mixte de recherche 970, PARCC, France; f Institut National de la Santé et de la Recherche Médicale, Unité mixte de recherche-S 769, Châtenay-Malabry, France; g Université Paris-Sud, Faculté de Pharmacie, Châtenay-Malabry, France; h SuperSonic Imagine, Aix-en-Provence, France; i Université Paris Descartes, Faculté de Médecine, Paris, France

**Keywords:** echocardiography, elasticity, imaging, ischemia, myocardial stiffness, myocardium, EDP, end-diastolic pressure, EDSSR, end-diastolic stress-strain relationship, LV, left ventricle/ventricular, SWI, shear wave imaging

## Abstract

**Objectives:**

The aim of this study was to investigate the potential of shear wave imaging (SWI), a novel ultrasound-based technique, to noninvasively quantify passive diastolic myocardial stiffness in an ovine model of ischemic cardiomyopathy.

**Background:**

Evaluation of diastolic left ventricular function is critical for evaluation of heart failure and ischemic cardiomyopathy. Myocardial stiffness is known to be an important property for the evaluation of the diastolic myocardial function, but this parameter cannot be measured noninvasively by existing techniques.

**Methods:**

SWI was performed in vivo in open-chest procedures in 10 sheep. Ligation of a diagonal of the left anterior descending coronary artery was performed for 15 min (stunned group, n = 5) and 2 h (infarcted group, n = 5). Each procedure was followed by a 40-min reperfusion period. Diastolic myocardial stiffness was measured at rest, during ischemia, and after reperfusion by using noninvasive shear wave imaging. Simultaneously, end-diastolic left ventricular pressure and segmental strain were measured with a pressure catheter and sonomicrometers during transient vena caval occlusions to obtain gold standard evaluation of myocardial stiffness using end-diastolic strain-stress relationship (EDSSR).

**Results:**

In both groups, the end-systolic circumferential strain was drastically reduced during ischemia (from 14.2 ± 1.2% to 1.3 ± 1.6% in the infarcted group and from 13.5 ± 3.0% to 1.9 ± 1.8% in the stunned group; p <0.01). SWI diastolic stiffness increased after 2 h of ischemia from 1.7 ± 0.4 to 6.2 ± 2.2 kPa (p < 0.05) and even more after reperfusion (12.1 ± 4.2 kPa; p < 0.01). Diastolic myocardial stiffening was confirmed by the exponential constant coefficient of the EDSSR, which increased from 8.8 ± 2.3 to 25.7 ± 9.5 (p < 0.01). In contrast, SWI diastolic stiffness was unchanged in the stunned group (2.3 ± 0.4 kPa vs 1.8 ± 0.3 kPa, p = NS) which was confirmed also by the exponential constant of EDSSR (9.7 ± 3.1 vs 10.2 ± 2.3, p = NS).

**Conclusions:**

Noninvasive SWI evaluation of diastolic myocardial stiffness can differentiate between stiff, noncompliant infarcted wall and softer wall containing stunned myocardium.

Assessment of diastolic left ventricular function is critical for the evaluation of heart failure and ischemic cardiomyopathy. Myocardial stiffness is thought to play a key role in diastolic function (1). In patients with preserved ejection fraction, abnormalities in left ventricle (LV) relaxation and LV stiffness are key pathophysiological mechanisms (2). Myocardial stiffness is also known to be a very strong prognosis parameter in hypertrophy (3) and dilated cardiomyopathy (4). In myocardial infarction, tissue Doppler and strain echocardiography are established methods for tracking myocardial deformation for the evaluation of systolic function 5, 6, 7. Few studies, however, have reported the use of these techniques to describe diastolic deformation, and none of the techniques are able to evaluate diastolic myocardial stiffness. However, diastolic myocardial stiffness changes appear very early during myocardial ischemia 8, 9. Moreover, several studies demonstrated an increase in myocardial stiffness after myocardial infarction using different techniques such as finite elements analysis (10) or stress-strain measurements (11). Pislaru et al. (12) demonstrated, using strain imaging, that passive diastolic myocardial deformation was correlated to the change in myocardial stiffness during myocardial ischemia. However, they emphasized that the magnitude of passive deformation was load-dependent contrary to indexes of myocardial stiffness. Thus, currently, myocardial stiffness cannot be quantified noninvasively by echocardiographic tools such as tissue Doppler or strain echocardiography.

Recently, we developed shear wave imaging (SWI), a new ultrasound-based technique for quantitatively mapping the stiffness of soft tissues characterized by using the Young modulus defined by the slope of the stress-strain curve. This technique belongs to the field of multiwave imaging as it combines 2 waves: a shear wave providing stiffness contrast and ultrasonic waves providing millimeter-level spatial resolution (13). The clinical potential of this approach has been recently demonstrated in the field of breast lesion imaging (14), as well as in liver (15) and arteries (16). In the field of cardiac imaging, we have already shown its potential for the evaluation of myocardial contractility (17) and fiber architecture (18).

The present study investigated the potential of SWI in vivo to noninvasively quantify changes in passive diastolic stiffness in an ovine model of ischemic heart failure in order to discriminate between infarcted myocardium and stunned myocardium. This is of upmost importance because in a clinical setting it is still challenging to differentiate between these 2 types of ischemia only by using strain echocardiographic evaluation of the regional active systolic function at rest.

## Materials and Methods

### Animal model

Animal procedures were approved by the Institutional Animal Care and Use Committee of Hôpital Européen Georges Pompidou (PARCC) according to the European Commission guiding principles (2010/63/EU). Ten sheep, weighing 49 ± 5 kg, were anesthetized with thiopental (0.5 ml/kg), intubated, ventilated at 15 ml/kg with 2% isoflurane, and given glycopyrrolate (0.4 mg intravenous) and vancomycin (0.5 g intravenous). A sterile lateral thoracotomy was performed. Vital signs, including heart rate, oxygen saturation, and arterial blood pressure, were monitored. The anterior wall at the middle left ventricular level was selected as the region of interest.

The 10 animals were divided into 2 different groups. In group I (n = 5), acute myocardial ischemia was induced by ligating 1 diagonal branch of the left anterior descending coronary artery for 15 min, followed by 40 min of reperfusion. In group II (n = 5), acute myocardial ischemia was induced for 120 min, followed by 40 min of reperfusion. The objective was to achieve infarction in order to compare the stiffness of infarcted myocardium (group II) with that of stunned myocardium.

### Myocardial stiffness measured by shear wave imaging

**Figure 1:**
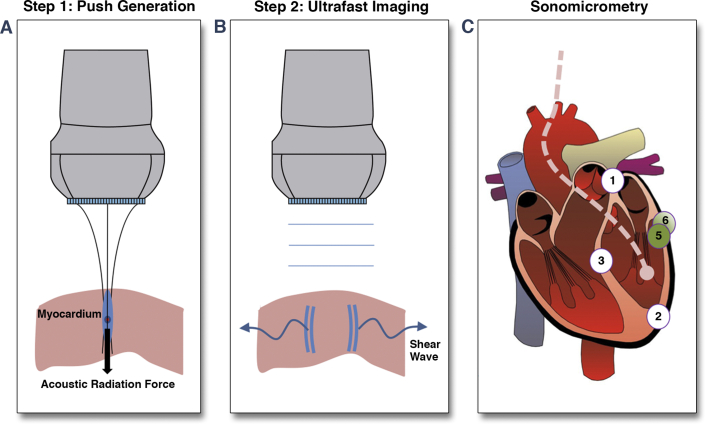
SWI Principle **(A)** Remote shear wave generation: an ultrasonic burst is focused on the myocardium. The acoustic radiation force generates tissue displacements at the focal zone. **(B)** Ultrafast imaging: Pulse plane waves are transmitted by the same ultrasonic probe at a repetition frequency of 10,000 Hz. The pulse echo signals are stored in a computer, and images are beam-formed offline. **(C)** Schematic representation of crystal implantation. A crystal pair (1 and 2) was implanted for measurement of LV long axis, a pair (3 and 4) for anteroposterior internal diameter measurement, and a pair (5 and 6) for segmental deformation (approximately 20 mm apart) in the **blue shaded** region (region at ischemic risk).

SWI is based on the remote generation of shear waves in soft tissue by acoustic radiation force combined with ultrasonic ultrafast imaging of the shear wave propagation, using the same ultrasonic transducer (19). A short burst (300 μs) of focused ultrasound was transmitted by a diagnostic ultrasonographic probe (linear array, 8-MHz central frequency; SuperSonic Imagine, Aix-en-Provence, France) to induce micrometric tissue displacements in a small zone of the myocardium due to acoustic radiation force (Figure 1A). In response to that transient mechanical excitation, a shear wave is generated in the low–kHz-frequency range and propagates in the myocardium at velocities from 1 to 10 m/s, depending upon tissue stiffness (Figure 1B). The originality of SWI consists of imaging the shear wave propagation at an ultrahigh frame rate (10,000 images/s) by using the same diagnostic probe connected to an ultrafast ultrasonic scanner (Aixplorer, SuperSonic Imagine). Tissue velocity maps were computed offline for each frame by using in-phase-quadrature frame to frame cross-correlation. Myocardial wall motion was removed by subtraction of the average wall motion during the acquisition, bringing to light tissue motion induced solely by the shear wave. The average wall motion was computed as the mean tissue velocity over the region of interest and over a window time of 4 ms after the shear wave generation (20). Shear velocity was computed at each depth of the image using spatiotemporal data from shear wave propagation. Finally, the shear modulus μ (i.e., stiffness) was derived at each location using the equation:

(Equation 1) $$\mu =\rho c^{2}$$

where *c* is the shear wave velocity, and *ρ* is the volume mass of the tissue. Equation 1 assumes the shear wave propagation to be isotropic, but this does not hold in the myocardium 18, 21. To overcome any anisotropic effect, the segment length was measured in the circumferential direction by the sonomicrometers (crystal pair 5 and 6), and the ultrasound probe was also aligned on the segment direction to quantify the shear wave propagation along the circumferential direction. In this configuration, midwall myocardial fibers are oriented mainly along the circumferential direction so that shear wave velocity measured in the midwall region provides an evaluation of circumferential stiffness. A region of interest 3 mm deep × 10 mm long centered on the midwall was automatically selected based on manual delineation of the endocardial and epicardial borders for each acquisition.

Stiffness measurements were repeated every 60 ms to quantify the dynamics of myocardial stiffness in real time. Fifteen acquisitions were performed, the total of which covered more than 1 cardiac cycle (∼600 ms). LV pressure and electrocardiography (ECG) were recorded at the same time on an external analog-to-digital board (Usbamp; gTec Medical Engineering, Schiedlberg, Austria), allowing synchronization of the acquisition in post-processing. The end-diastolic time point was set at the onset of the QRS complex.

### Segment length measurements

Measurements of segment length were performed using a set of 2-mm sonomicrometer crystals (Sonometrics, London, Ontario, Canada). Two pairs of crystals were implanted near the endocardium for LV volume measurement. The first pair consisted of a crystal implanted at the base and the other at the apex of the LV for measuring the long axis *LA* (Figure 1C). The second pair measured the short axis, *SA*: the anteroposterior LV diameter at the mid-ventricular level. Two other crystals were implanted mid-wall at the mid-ventricular level of the anterior wall within the suspected ischemic region in order to measure the segment length in the circumferential direction. The segmental strain was computed as

(Equation 2) $$\varepsilon_{L}=\frac{L-L_{0}}{L_{0}}$$

where *L*_*0*_ is the end-diastolic segment length at baseline (crystals 5–6). The end-diastolic thickness of the imaged wall was measured from the echocardiographic images recorded during the SWI acquisitions according to the latest recommendations of American Society of Echocardiography.

### Evaluation of end-diastolic stress-strain relationship

In current approaches, precise determination of passive myocardial elastic modulus requires measurement of end-diastolic stress-strain relationship. Strain was measured by sonomicrometry, whereas myocardial stress cannot be measured in vivo but only estimated by use of a geometrical model. A thick walled spherical model described by Mirsky and Parmley (22) was assumed here in order to compute midwall circumferential stress from LV pressure measurements as follows

(Equation 3) $$\sigma_{\theta }=Pa^{3}\left(1+4b^{3}/{\left(a+b\right)}^{3}\right)/\left(b^{3}-a^{3}\right)$$

where *a* and *b* are the internal and external radii, respectively, and *P* is the LV ventricular pressure. The term *b* was computed as the sum of *a*, and the thickness was measured by echocardiography.

End-diastolic segmental strain was plotted against end-diastolic circumferential stress for several cardiac cycles during vena cava occlusions. The diastolic stress-segmental strain relationship was approximated by the exponential equation σ = Ae^βε^, where σ is the diastolic mid-wall circumferential stress, ε is the diastolic circumferential strain, and *A* and β are curve-fitting constants used to quantify passive stiffness (22). Pressure was measured using a 5-F micromanometer Millar catheter (model MPR-500, Millar Instruments Inc., Houston, Texas) placed inside the LV. Temporary inferior vena cava occlusions were induced to obtain the stress-strain relationship over a large range of end-diastolic pressure values.

### TTC staining

The heart was explanted and sectioned into short-axis slices approximately 5 mm thick. The tissue was placed into 1% 2,3,5-triphenyl tetrazolium chloride (TTC) in phosphate buffer and incubated for 20 min at 37°C. The slices were then inspected visually to identify the presence of pale white infarcted tissues.

### Statistical analysis

Values are mean ± SD. Changes in myocardial stiffness were analyzed using a paired 2-tailed Student *t* test to evaluate the significance of differences between individual mean values under different inotropic effects or after occlusion-reperfusion. When there was more than 1 comparison between means, ANOVA analysis was performed. If a significant trend was found by the ANOVA test, a Newman-Keuls test was performed to compare 2 different means. Statistical significance was inferred for a p value <0.05. Linear regression was used for correlation between systolic stiffness and contractility.

## Results

### End-diastolic myocardial stiffness of infarcted myocardium

At baseline, end-diastolic SWI myocardial stiffness in group I (1.8 ± 0.3 kPa) was similar to that in group II (1.7 ± 0.4 kPa) as was stiffness constant measured by sonomicrometry (9.7 ± 3.1 and 8.8 ± 2.3, respectively) and systolic segmental deformation (13.5 ± 3% and 14.2 ± 1.2%, respectively) (Table 1). During coronary occlusion, segmental systolic myocardial function measured as the percentage of segment shortening (%ΔL) was strongly and significantly reduced in both groups (1.9 ± 1.8%; p < 0.01 in group I; and 1.3 ± 1.6%; p < 0.01 in group II). At the end of the ischemic period, SWI diastolic stiffness increased drastically in group II after 120 min (6.2 ± 2.2 kPa; p < 0.05) but not in group I after 15 min (2.1 ± 0.6 kPa, p = NS). During reperfusion, the reduction of segmental systolic function persisted in group II (1.3 ± 1.7%; p < 0.01) and recovered partially in group I (6.2 ± 4.7%; p < 0.01). After reperfusion, SWI diastolic stiffness increased even more in (infarcted) group II (12.1 ± 4.2 kPa; p < 0.01) but not in (stunned) group I (2.3 ± 0.4 kPa, p = NS). These results were confirmed by the increase in stiffness constant as measured by sonomicrometry in group II (25.7 ± 9.5; p < 0.05), whereas it remained unchanged in group I (10.2 ± 2.3, p = NS). No significant differences were found among end-diastolic pressure, end-systolic pressure, and heart rate at reperfusion. TTC staining of explanted myocardium confirmed the presence of a large infarcted zone for all animals in group II and the absence of infarcted zone for all animals in group I.

**Table 1:** Effect of Ischemia-Reperfusion on Systolic and Diastolic Myocardial Function Measured by Ultrasonic Crystals and on End-Diastolic Stiffness Measured by SWI

Stunned Group (I) (n = 5)	Baseline	After 15 min of Occlusion	After 30 min of Reperfusion	p Value
Heart rate, beats/min	112.0 ± 13.0	106.0 ± 13.0	106.0 ± 11.0	NS
EDP, mm Hg	13.5 ± 3.0	14.5 ± 1.9	12.5 ± 2.3	NS
ESP, mm Hg	68 ± 11.3	62.1 ± 7.8	61.7 ± 11.8	NS
%ΔL	13.5 ± 3.0	1.9 ± 1.8†	6.2 ± 4.7†‡	<0.01
Stiffness constant (crystals)	9.7 ± 3.1	Not evaluated	10.2 ± 2.3	NS
SWI ED stiffness, kPa	1.8 ± 0.3	2.1 ± 0.6	2.3 ± 0.4	NS

Infarcted Group (II) (n = 5)	Baseline	After 2 h of Occlusion	After 30 min of Reperfusion	p Value
Heart rate, beats/min	118.0 ± 14.0	113.0 ± 37.0	110.0 ± 26.0	NS
EDP, mm Hg	13.1 ± 2.2	13.5 ± 5.9	14.7 ± 3.5	NS
ESP, mm Hg	72.0 ± 8.2	54.0 ± 14.2∗	55.9 ± 14.5∗	<0.05
%ΔL	14.2 ± 1.2	1.3 ± 1.6†	1.3 ± 1.7†	<0.01
Stiffness constant (crystals)	8.8 ± 2.3	Not evaluated	25.7 ± 9.5∗	<0.05
SWI ED stiffness, kPa	1.7 ± 0.4	6.2 ± 2.2∗	12.1 ± 4.2†‡	<0.01

Values are mean ± SD.

%ΔL = [(end-diastolic length – end-systolic length)/(end-diastolic length)].

EDP = end-diastolic pressure; ESP = end-systolic pressure; NS = nonsignificant; SWI = shear wave imaging.

∗ p < 0.05 with control.

† p < 0.01 with control.

‡ p < 0.01 with ischemia.

**Figure 2:**
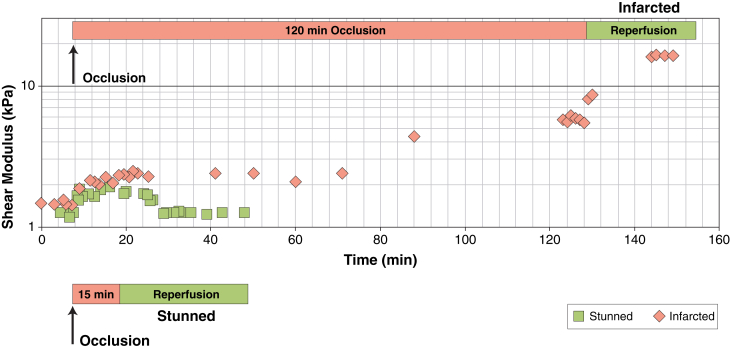
Variation of the Myocardial Stiffness Measured by SWI During Ischemia Reperfusion The variation of SWI myocardial diastolic stiffness during occlusion and reperfusion is shown on 1 animal from the stunned group **(green)** and 1 from the infarct group **(pink)**.

Figures 2A and 2B shows the evolution of myocardial stiffness measured by SWI during occlusion and reperfusion in 1 animal of group I and 1 of group II. In group I (Figure 2A), the end-diastolic stiffness of stunned myocardium (group I) was unchanged after reperfusion. In the animal of group II, in contrast, diastolic myocardial stiffness increased from 1.7 kPa to 4.8 kPa after 120 min of occlusion. After reperfusion, the stiffness of the infarcted myocardium increased even more and reached 16.4 kPa.

**Figure 3:**
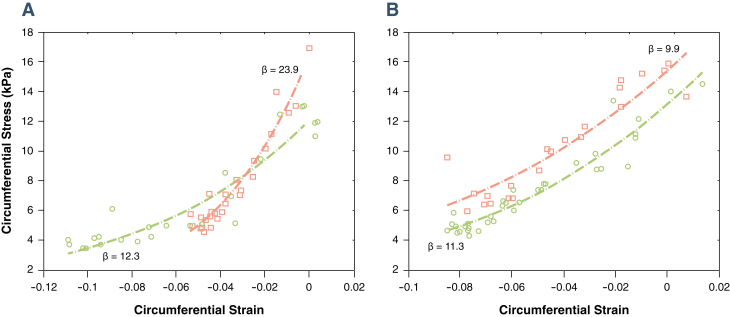
End-Diastolic Strain-Stress Relationship Before and After Infarction, Obtained by Sonomicrometry The end-diastolic strain-stress relationship is shown for 1 animal from each group. In the infarcted myocardium group **(A)**, the exponential constant of the stress-strain relationship increased strongly after ischemia reperfusion whereas in the stunned group **(B)**, the exponential constant did not change after ischemia reperfusion **(pink)** compared with baseline **(green)**.

**Figure 4:**
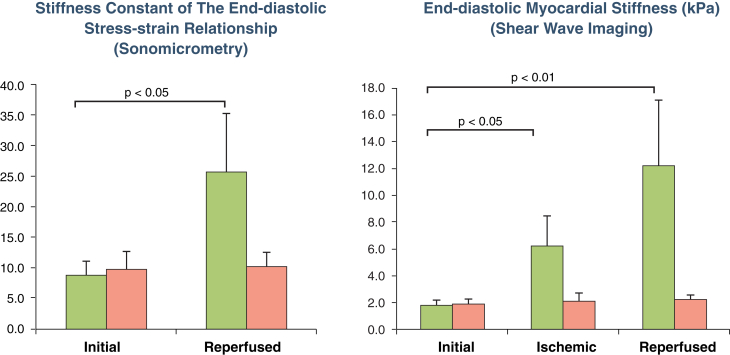
Myocardial Stiffness After Ischemia-Reperfusion Myocardial stiffness is measured by sonomicrometry **(left)** and SWI **(right)** in stunned **(pink)** and infarcted **(green)** animals.

Moreover, the diastolic stiffness constant measured invasively by sonomicrometry confirmed that end-diastolic stiffness increased strongly in infarcted myocardium and was unchanged in stunned myocardium (Figure 3). Figure 4 compares the stiffness changes measured by SWI and that by sonomicrometry for all 10 animals and shows that end-diastolic myocardial stiffness differs drastically in stunned and infarcted myocardium, using both techniques.

## Discussion

This is the first study to quantify diastolic stiffness during myocardial ischemia by ultrasonography imaging and without the need of invasive sensors such as pressure catheters or sonomicrometer crystals. We demonstrated that reperfused infarcted myocardium has markedly increased diastolic myocardial stiffness which persisted after reperfusion. In addition, we showed that stunned myocardium preserved tissue compliance with no significant change of diastolic myocardial stiffness. As opposed to stiffness, regional systolic myocardial function significantly decreased in both groups.

In contrast to systole, for which there is a gold standard (end-systolic elastance) that can be obtained relatively easily, there is no universally accepted method for evaluating diastolic myocardial stiffness. During diastole, the myocardium becomes completely passive when relaxation is fully completed, and it was established in the early 1970s that passive properties of the myocardium could be defined by 3 parameters: stress, strain, and tangent modulus (elasticity or stiffness) (22). Different means for calculating the stiffness modulus have been proposed. We chose a relatively simple index that has been previously published (22) in which the coefficient β that quantifies passive stiffness is obtained by curve fitting of an exponential equation, σ=Ae^βε^, relating the circumferential stress and strain term ε. This allowed us to compare diastolic stiffness measured by LV pressure and segment lengths from ultrasonic crystals with the end-diastolic stiffness measured by the SWI technique.

SWI appears thus to be a valuable noninvasive technique in the evaluation of diastolic regional function which could be useful in the evaluation of regional ischemia. As described long ago with sonomicrometry, coronary occlusion in-vivo induced an immediate bulging of the ischemic segment with its net systolic shortening becoming close to zero or negative both in dogs (23) and in baboons (24). These results were again corroborated with ultrasonic crystals in sheep in the present study. The percentage of systolic shortening recovered partially after 30 min of reperfusion when the occlusion had a 15-min duration (from 13.5% at baseline to 1.9% during ischemia and 6.2% during reperfusion) (Table 1), but no recovery was observed when the duration of ischemia was 2 h. The partial recovery of myocardial function observed after reperfusion when the ischemic period was 15 min corresponds to the well-known phenomenon of “myocardial stunning” (25). It has been described as a prolonged post-ischemic contractile dysfunction of myocardium salvaged by reperfusion. The mechanism of stunning involves generation of oxygen radicals, alteration in calcium homeostasis, and possibly, alterations in protein structure.

In this study we have shown that a significant stiffening of the myocardium at end-diastole was observed in the infarcted group after 120 min of acute ischemia which further increased after 30 min of reperfusion. In contrast, passive myocardial stiffness was unchanged in the stunned group after reperfusion (Table 1, Figure 4). Although systolic deformation was strongly reduced in both groups, diastolic myocardial stiffness increased only in the infarcted group. These modifications of systolic and diastolic function are in line with previously published results in both experimental animals and in humans 12, 26, 27.

### Clinical application

We can expect to use SWI in a post-myocardial infarction clinical setting to decide upon revascularization. Revascularization of significant coronary artery disease in a patient with LV dysfunction and symptomatic heart failure has been associated with improved cardiac function and cardiovascular outcomes (28). The accurate assessment of myocardial viability is crucial to guide treatment and predict the benefit of coronary revascularization 29, 30. One can envision that region with increased diastolic myocardial stiffness may not be viable and would not recover after coronary revascularization. This needs to be validated with a viability imaging modality such as positron emission tomography scan.

### Study limitations

Open-chest experimental and sonomicrometry crystals may affect myocardial deformation. Moreover, shear wave propagation may be affected by the particular boundary conditions of the open chest configuration. Specific boundary conditions of transthoracic imaging but also when fluids accumulate around the heart such as in pericardial or pleural effusion may affect the propagation of shear waves, and these particular situations need to be investigated further. For validation purpose, we used a simplistic thick-wall spherical model to compute the wall stress and evaluate the diastolic stiffness constant from the stress-strain relationship. In this study we chose 2 extremes (stunned and infarcted) to validate a concept. More studies are necessary to relate myocardial stiffness to infarct transmurality.

## Conclusions

In this study, we quantitatively assessed in vivo the passive myocardial stiffness using SWI in normal, stunned, and infarcted myocardium. SWI appears thus to be a valuable noninvasive technique in the evaluation of diastolic stiffness without the need of associated LV pressure measurement. A strong stiffening of the myocardium was found in diastole after infarction whereas end-diastolic myocardial stiffness remained unchanged in stunned myocardium. End-diastolic stiffness measured by SWI can thus also be used to determine the passive diastolic properties of ischemic/reperfused myocardium in an open chest configuration. The application of SWI to transthoracic imaging remains to be demonstrated in further studies.

Perspectives **COMPETENCY IN MEDICAL KNOWLEDGE:** Myocardial stiffness is a major determinant of ventricular function both in heart failure and during ischemia. This parameter cannot be measured noninvasively by existing techniques. Successful evaluation of passive diastolic myocardial stiffness in vivo using noninvasive ultrasonography-based SWI can discriminate between stunned and infarcted myocardium, opening the door to noninvasive evaluation of myocardial viability.

**TRANSLATIONAL OUTLOOK:** Noninvasive evaluation of myocardial stiffness by SWI still needs to be confirmed in human patients. We are currently starting a human study of normal volunteers and heart failure patients to validate this parameter in a clinical setting. Further study with longitudinal follow-up will be required to test the prognostic value of this new noninvasive parameter.
